# The role of hormonal markers in predicting reproductive lifespan in girls with Turner syndrome—a retrospective study

**DOI:** 10.1210/jendso/bvag122

**Published:** 2026-06-05

**Authors:** Sanne van der Coelen, Theo Sas, Annette Richter-Unruh, Didi Braat, Kathrin Fleischer, Sapthami Nadesapillai, Joanna IntHout, Janielle van der Velden

**Affiliations:** Department of Obstetrics and Gynecology, Radboud University Medical Center, Nijmegen 6500 HB, the Netherlands; Department of Pediatrics, Erasmus University Medical Center, Sophia Children's Hospital, Rotterdam 3015 GD, the Netherlands; Center for Pediatric and Adult Diabetes Care and Research, Rotterdam 3011 TA, the Netherlands; Department ofChildren and Adolescent Endocrinology MVZ Eberhard, Dortmund 44139, Germany; Department of Obstetrics and Gynecology, Radboud University Medical Center, Nijmegen 6500 HB, the Netherlands; Department of Reproductive Medicine, Nij Geertgen Center for Fertility, Elsendorp 5424 SM, the Netherlands; Department of Obstetrics and Gynecology, Radboud University Medical Center, Nijmegen 6500 HB, the Netherlands; IQ Health Science Department, Radboud University Medical Center, Nijmegen 6500 HB, the Netherlands; Department of Paediatrics, Amalia Children's Hospital, Radboud University Medical Center, Nijmegen 6500 HB, the Netherlands

**Keywords:** Turner syndrome, ovarian function, prediction model, premature ovarian insufficiency, follicle stimulating hormone, antimüllerian hormone

## Abstract

**Context:**

Early prediction of ovarian reserve is essential to improve fertility counseling in girls with Turner syndrome (TS).

**Objective:**

This work aimed to examine the occurrence of puberty and longitudinal hormone patterns in girls with TS, and to take an initial step toward developing a prediction model for ovarian function. Main outcome measures included thelarche, menarche, and age of premature ovarian insufficiency (POI).

**Methods:**

A retrospective cohort study was conducted of girls with TS. Levels of serum follicle-stimulating hormone (FSH), luteinizing hormone (LH), antimüllerian hormone (AMH), inhibin B, and information on karyotype and puberty were extracted from the medical records. A joint model combining a Cox survival model and 2 linear mixed-effects models for FSH and AMH was used to model the association between POI and longitudinal hormone values.

**Results:**

A total of 365 girls were included, of whom 287 had available data on pubertal development. In this patient cohort, 43% had monosomy 45,X, 17% had mosaic 45,X/46,XX, 18% had an isochromosome X, and 22% had an “other” karyotype in lymphocytes. Girls with spontaneous puberty had lower FSH and higher AMH before age 8 years compared to girls without spontaneous puberty. The joint model showed that lower AMH is significantly related to POI, while FSH was not significantly related to POI. The individual predicted risks on POI come with large uncertainty.

**Conclusion:**

AMH and FSH patterns contribute to understanding ovarian reserve at an early age in girls with TS. Creating a prediction model remained challenging and more data are needed.

Turner syndrome (TS) in females involves the absence or alteration of one of the sex chromosomes. Females with TS commonly exhibit a reduced ovarian reserve. The complete depletion of ovarian reserve occurs for some girls already at birth, for two-thirds before puberty, and only 2% to 14% still have a sufficient ovarian reserve at the time they intend to become pregnant [1, 2]. To enhance the prospects of biological offspring, the option of fertility preservation may be pursued during childhood or adolescence. Contemporary techniques for fertility preservation include oocyte vitrification and ovarian tissue cryopreservation (OTC). However, oocyte vitrification is limited to girls who have spontaneous menstrual bleeding and who are psychologically mature to undergo the process of ovarian stimulation and oocyte aspiration [3]. OTC is a technique that can be performed before puberty. OTC requires invasive surgery to remove and preserve one of the ovaries, and it is still considered experimental for girls with TS as its efficacy has not yet been proven [4, 5]. Counseling is needed to weigh the benefits and risks of these procedures [6, 7]. Nonetheless, counseling is challenging because of the uncertainty of ovarian reserve decline in an individual girl with TS.

Hormone levels during childhood might foresee whether spontaneous puberty occurs. However, the added value of gonadotropin measurements during childhood is limited by the quiescent hypothalamus-pituitary-gonadal axis. Some studies have suggested that spontaneous puberty is less likely if follicle-stimulating hormone (FSH) levels during childhood exceed the upper limit of reference [8, 9]. On the contrary, other studies have indicated that gonadotropins in girls with TS are often within the normal range during childhood, both in girls with and without spontaneous puberty [10, 11]. Another marker often used to estimate ovarian reserve is antimüllerian hormone (AMH). AMH is produced by the growing pool of follicles and thereby reflects the ovarian reserve [12]. Girls with TS aged 12 years and older with AMH levels above the detection limit were 19 times more likely to have spontaneous thelarche compared to girls with AMH below the detection limit [13, 14]. In addition, undetectable levels of AMH were associated with absent puberty and were more often seen in girls with a monosomic karyotype [15, 16]. While several studies have used AMH to predict puberty, achieving precise predictions of ovarian reserve decline and, consequently, the timing of premature ovarian insufficiency (POI) in individual girls based on AMH levels proved difficult to attain [15].

Current knowledge based on various hormonal parameters, karyotype, and age is insufficient to estimate the probability of spontaneous puberty onset or the continuation of ovarian function later in life. This limitation arises because most research relies on cross-sectional data, longitudinal measurements of only one hormone, or small cohort sizes.

This study provides an overview of pubertal development and longitudinal hormone levels in a cohort of girls and women with TS and correlates the patterns to ovarian function in terms of spontaneous thelarche, menarche, and the timing of POI. With these results, we aim to initiate a first step toward developing a prediction model for ovarian reserve. The results can be used by health-care providers to support counseling of girls with TS regarding individualized family planning options.

## Materials and methods

This descriptive retrospective cohort included girls with TS in the Netherlands (University Medical Center Radboudumc Nijmegen and Erasmus MC Rotterdam) and Germany (Medizinisches Versorgungszentrum Dortmund). Data of sex hormones and pubertal development were obtained from the medical records from January 1, 1960, through March 1, 2022. Data are presented according to the STROBE guidelines (Supplementary Table S1 [17]).

All patients gave informed consent to the hospital to use their data for research. The Radboudumc Ethical Review Board (2020-6800) considered that no individual informed consent was needed.

### Study population

The electronic patient files of 626 girls and women with TS were screened. There was no age confinement or minimum follow-up period. The diagnosis of TS was based on cytogenetic analysis of 30 or more cells from peripheral blood cultures with at least 10% aneuploidy [7]. Girls were excluded if no hormone measurements were described in the medical record (n = 254), if the diagnosis of TS was not conform the karyotypes described in the TS guidelines (n = 5), or if they were assigned male at birth (n = 2). Measurements of a girl taken after the start with estrogens, such as puberty induction or hormone replacement therapy, or after ovariectomy, were excluded from the analysis.

### Karyotype

Participants were divided into subsets solely based on their karyotype analyzed in lymphocytes. This included monosomy (45,X), mosaic-45,X/46,XX, isochromosome (46,X,i(X) and 46,X,idic(X)), and other karyotypes (eg, 45,X/46,XX/47,XXX, ring-X-chromosome, deletion, duplication, Y-material). Isochromosome X was analyzed separately from the group with other structural aberrations because it is the third common genotype in girls with TS. The genotype in buccal cells was included in the database if available, but was not used in the analysis of ovarian function due to the limited availability of buccal cell data.

### Pubertal development

Pubertal development was described according to Tanner stages, in which Tanner B1 was assigned prepuberty, and Tanner B2 was assigned as spontaneous onset of thelarche. POI was defined by hypergonadotropic levels (FSH ≥ 25 U/L, ie, twice the upper reference limit), or initiation of female hormone therapy by the clinician for puberty induction or due to signs of POI/depleted ovarian reserve, such as flushes or irregular menstrual cycle, or increased FSH levels.

### Hormone assays

Hormone measurements of FSH, luteinizing hormone (LH), AMH, and inhibin B were collected. Various assays were used across clinics from 1960 to 2022. The vast majority of the analyses were conducted in the last 20 years, during which the following assays were used: FSH and LH were analyzed with the Elecsys method on the Cobas E801 system (Roche Diagnostics) in Radboudumc, and with Immunoassay, Siemens Atellica IM 1300 in ErasmusMC with lower limit of detection (LLOD) of 0.30 U/L. AMH and inhibin B were analyzed with a chemiluminescence immunoassay (Access) and an enzyme-linked immunosorbent assay (Gen II) (Beckman Coulter, RRID:AB_2827405) in Radboudumc and ErasmusMC, with an LLOD 0.10 µg/L and 10 ng/L respectively. The total imprecision of AMH is 5.6% and 4.2% at 0.93 μg/L and 5.35 μg/L, respectively. The total imprecision of inhibin B is 10.1% and 7.5% at 47.1 ng/L and 250.1 ng/L, respectively. A serum level measured below the LLOD was converted into LLOD/2 to correct for distortion of the data distribution.

### Ovarian status

The ovarian status was assigned to each girl's measurement based on Tanner stage and serum FSH. Measurement distribution by ovarian status included i) prepubertal: Tanner B1 without signs of POI and FSH less than 25 U/L (age >4 years) [18]; ii) prepubertal POI: puberty induction (Tanner B1 and start of female hormones) or Tanner B1 and hypergonadotropic serum FSH greater than or equal to 25 U/L; iii) ongoing ovarian function: Tanner greater than or equal to B2 and FSH less than 25 U/L; and iv) POI after spontaneous thelarche and/or menarche: Tanner greater than or equal to B2 and FSH greater than or equal to 25 U/L.

### Statistical analysis

Girls’ clinical characteristics are presented using descriptive statistics for the total number of participants, per karyotype group, and per ovarian status. Continuous variables are presented as medians and interquartile ranges (IQR). Categorical variables are presented with counts and percentages. Sensitivity, specificity, and area under the receiver operating characteristic curve (AUC) are calculated to assess the discriminative value of maximum FSH levels in the age range 4 to 8 years to predict spontaneous thelarche and the discriminative value of measurable AMH in the age range of 8 to 12 years to predict spontaneous menarche. Only hormone measurements obtained prior to the occurrence of thelarche or menarche are included in the statistical analysis.

Trends in longitudinal hormones are displayed graphically using spaghetti plots on a logarithmic scale for the subject-specific longitudinal trajectories over time, along with a fitted mean and 95% CI using locally estimated scatterplot smoothing (loess) for puberty-specific categories. Lack of overlap of the CIs is interpreted as a statistically significant difference at a level of 5%. In case of small overlap of the 95% CI, the overlap of the 90% CIs was checked. If these did not overlap, it is interpreted as a statistically significant difference at a level of 5% [19]. Kaplan-Meier curves are presented to visualize the probability of ongoing ovarian function during the follow-up period, conditional on karyotype (mosaic-45,X/46,XX vs all other karyotypes, referred to as miscellaneous karyotypes), and the AMH level at onset of puberty (age 10-13 years).

A Bayesian joint model for longitudinal and time-to-event outcomes was used to combine the longitudinal hormone trajectories to predict the risk of POI. Joint modeling is a statistical technique that combines several submodels, in this case i) 2 linear mixed-effects models estimating the trajectories of the 2 longitudinal predictors (ln(FSH) and ln(AMH)) over age, with random intercept and slope per participant, and ii) a Cox proportional-hazards survival model to predict the risk of the dichotomous outcome (POI) by age. Joint models are very suitable to model the relation between endogenous markers (such as FSH and AMH) and a related event, in this case POI after thelarche. The marker levels and the event can influence each other in both directions, for example, by feedback loops, are measured with error, and the complete history is often not available; consequently, directly using the marker values in the survival model can underestimate the predictive value of the markers. A joint model estimates the true evolution of the ln(FSH) and ln(AMH) values, and uses these, for example at age 12, to predict the probability of POI after thelarche from age 12 until 18 years. As the joint model is based on the hormone-trajectories by age, it allows for early risk prediction as well as for updating the predicted risk when new hormone measurements become available. For the 2 longitudinal models, available measurements from age 4 up to and including 30 years were included, using only values that were measured before POI after thelarche. The Akaike information criteria (AIC) of models with age, age^2^ and age^3^ were compared, and the model with the lowest AIC was selected. In case of similar AIC scores, the simplest model was selected. Both ln(FSH) and ln(AMH) could be modeled with only age and did not need a higher-level term for age. The final dynamic predictive performance of the joint model was assessed by using measurements up to age 12 to predict POI after thelarche at age 18 years. These cutoff ages were selected because determining the likelihood of POI at age 18 years can be valuable for counseling on fertility preservation options, as oocyte vitrification is generally recommended from this age onward. Sensitivity, specificity, and AUC, reflecting the model's ability to discriminate between girls with and without POI at a certain age (internal validation based on bootstrapping), and other characteristics of the joint model are reported.

Data retrieved from the medical records were stored using the online clinical trial platform Castor EDC (version 2021.6.2). Statistical analyses were performed with IBM SPSS Statistics for Windows (v25.0 IBM Corp [20]) and the open-source statistical analysis software R (v4.4.1; R Core Team 2024) and RStudio [21, 22]. The Bayesian joint model was fitted with the R package JMbayes2 (v0.5-7 [23]). A 2-sided *P* value less than .05 was considered statistically significant.

## Results

The study included 365 females with TS born between 1964 and 2019 (IQR 1999-2009). Females had a median of 3 visits per female (IQR 2-6 visits, cumulative count of 1428 visits). A total of 108 females had only 1 visit. The other 257 females had their first visit between ages 0.02 and 36.0 years (median 9.3, IQR 5.9-10.8 years), and their last visit before POI or end of study between ages 1.2 and 41.9 years (median 12.1, IQR 11.0-14.4 years), with a median follow-up period of 3.1 years (IQR 1.5-6.3 years).

The characteristics of the cohort, grouped according to karyotype in lymphocytes and pubertal phase, are provided in Table 1 and Supplementary Fig. S1 [17]. The genotype in buccal cells was analyzed in 140 females (38%). This resulted in a different combined karyotype in 18 of 140 females (13%): a 46,XX cell line in buccal cells of 12 females with a monosomy karyotype in lymphocytes, a 45,X/46,XX/47,XXX cell line in buccal cells in 3 females with a monosomy karyotype in lymphocytes, and a Y-chromosome in 3 females with a monosomy karyotype. All subsequent results are categorized according to karyotype in lymphocytes. Bilateral ovariectomy before age 12 years was performed in 8 girls; all had a Y-cell line. Visits after bilateral ovariectomy were excluded.

**Table 1:** Ovarian function of girls with Turner syndrome according to karyotype in lymphocytes

	Total	45,X	45,X/46,XX	i(X)	Other*^a^*
**Total**	**365**	**158** (**43)**	**63** (**17)**	**64** (**18)**	**80** (**22)**
**Prepubertal**	**78** (**21)**	**29** (**18)**	**10** (**16)**	**19** (**30)**	**20** (**25)**
Bilateral gonadectomy*^b^*	8	1	0	4	3
OTC	19	7	3	3	6
**Pubertal**	**287**	**129**	**53**	**45**	**60**
Prepubertal POI	169 (59)	111 (86)	8 (15)	27 (60)	23 (38)
Spontaneous thelarche	118 (41)	18 (14)	45 (85)	18 (40)	37 (62)
Spontaneous menarche	79 (28)	8 (6)	36 (68)	8 (18)	27 (45)
POI after thelarche	48 (17)	12 (9)	3 (6)	16 (36)	17 (28)
Before menarche	26	8	1	9	8
After menarche	22	4	2	7	9
Continued ovarian function	70 (24)	6 (5)	42 (79)	2 (7)	20 (33)
Before menarche	13	2	8	2	1
After menarche	57	4	34	0	19
**Age in years at:**					
Last monitoring prepubertal	8.7 (5.6-10.2)	8.5 (5.9-10.8)	8.4 (6.0-10.7)	7.1 (2.4-10.0)	9.0 (4.9-9.7)
Spontaneous thelarche	11.8 (10.5-12.9)	12.3 (10.6-13.3)	11.0 (9.9-12.5)	12.1 (11.3-13.6)	11.9 (10.9-12.8)
Spontaneous menarche	13.0 (12.1-13.8)	13.0 (11.2-14.1)	13.1 (12.1-14.1)	13.1 (11.6-13.9)	12.9 (12.0-13.5)
POI after thelarche	15.3 (14.0-17.0)	14.4 (12.9-16.7)	16.7 (NA)	15.4 (14.1-16.8)	16.2 (14.9-21.0)
Last monitoring without POI	15.4 (12.8-19.3)	14.6 (12.5-23.4)	15.1 (13.4-18.9)	12.7 (NA)	17.1 (12.5-20.7)

Frequencies are presented with number (percentage). Ages are presented as median (25th-75th percentile).

Abbreviations: OTC, ovarian tissue cryopreservation; POI, premature ovarian insufficiency.

^*a*^The group other included 21 girls with X-deletion, 24 girls with ring-X-chromosome, 14 girls with polyploidy, 12 girls with Y-material, 9 girls with translocation, and 3 girls with unclassified karyotype.

^*b*^Y-chromosome was detected in all girls with bilateral gonadectomy, in either lymphocytes or buccal cells.

LH was not analyzed statistically in relation to pubertal development, as FSH showed greater predictive value. LH trends across childhood are shown in Supplementary Fig. S1 [17]. Inhibin B is presented descriptively due to limited data availability.

### Prepuberty

At the end of follow-up, the occurrence of spontaneous thelarche and menarche was undetermined for 78 girls due to their current young age. These prepubertal girls were included in the study at a median age of 5.3 years (IQR 2.8-8.9) and were 8.7 years (IQR 5.6-10.2) at the last visit. Supplementary Fig. S2 [17] shows that gonadotropin levels exhibited a similar pattern in all karyotype groups: Levels were higher from 0 to 4 years, decreased between 5 and 10 years, and rose again after age 10. In general, girls with mosaic-45,X/46,XX had a trend toward lower FSH and LH concentrations and higher AMH concentrations than other karyotype groups. AMH was analyzed in 57 girls during prepuberty, showing consistent undetectable levels in 46 girls (81%). The other 11 girls had AMH levels between 0.10 and 4.7 µg/L (median 0.31, IQR 0.12-3.2 µg/L). Inhibin B was below the detection limit in 90% of the 43 prepubertal visits.

### Thelarche

**Figure 1:**
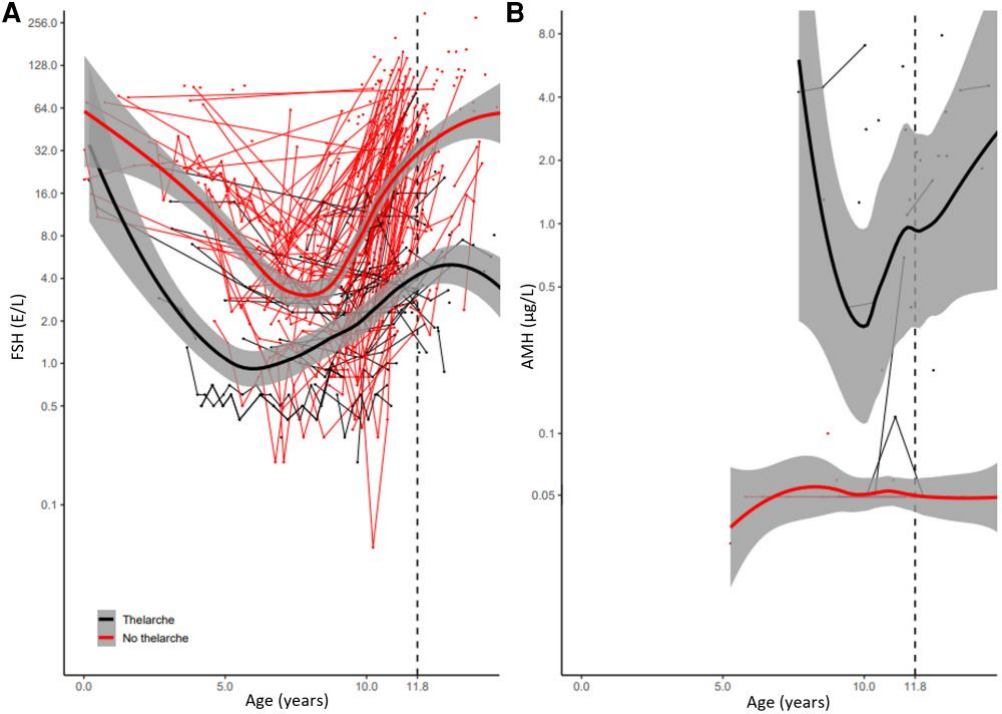
Longitudinal follicle-stimulating hormone (FSH) and antimüllerian hormone (AMH) levels and the occurrence of spontaneous thelarche. Each dot represents a hormone measurement, and consecutive measurements from the same girl are connected by a line. FSH and AMH levels are presented on a logarithmic scale. The gray area represents the fitted mean and 95% CI for puberty-specific categories. No overlap between the 95% CIs suggests a statistically significant difference. The vertical dotted line at age 11.8 years represents the mean age at spontaneous thelarche. In A, 75 girls with thelarche and 148 girls without thelarche were included. In B, 43 girls with thelarche and 66 girls without thelarche were included.

Spontaneous thelarche occurred in 118 of 287 girls (41%) at a median age of 11.8 years (IQR 10.5-12.9 years) (see Table 1). The proportion of girls with spontaneous thelarche varied between 14% in girls with a monosomy karyotype and 85% in girls with a mosaic-45,X/46,XX karyotype. Girls with a spontaneous thelarche had significantly lower FSH levels during childhood compared to girls without spontaneous thelarche (Fig. 1A), although this could not be confirmed during the first year of life (Supplementary Fig. S3 [17]). Specifically, girls with thelarche had lower FSH levels between 4 and 8 years (n = 19, maximum FSH 2.8 U/L, IQR 1.3-9.0 U/L) compared to FSH levels of girls without spontaneous thelarche (n = 50, maximum FSH 8.2 U/L, IQR 3.7-28.4 U/L). The AUC was 0.73 with the optimal cutoff identified by Youden's index was 3.1 U/L, yielding a sensitivity of 0.58, specificity of 0.80, and a Youden's index of 0.38, indicating limited discriminative ability.

Girls with spontaneous thelarche had more often detectable and higher levels of AMH, compared to girls without thelarche (Fig. 1B). AMH before age 8 years (and before thelarche) was measured in 1 girl with spontaneous thelarche (AMH 4.23 µg/L at age 7.7) and 3 girls with induced puberty, all of whom had AMH levels below the detection limit. Girls with thelarche (and who had hormone measurements obtained prior to its onset) aged 8 to 12 years had measurable AMH in 27 out of 34 (79%) girls, with a maximum AMH of 1.30 µg/L (IQR 0.10-3.20). In contrast, only 5 out of 59 girls (8%) without spontaneous thelarche had detectable AMH, with a maximum AMH of 0.10 µg/L (IQR 0.10-0.60), and all 5 had FSH levels greater than 25 U/L. Inhibin B was detectable in 9 out of 11 girls with spontaneous thelarche, with levels ranging from 17.0 to 133.2 ng/L, as opposed to only 1 out of 19 girls without thelarche (12 ng/L) (data not shown).

At the end of the study, 13 girls showed no symptoms of POI and had not yet experience menarche. These girls had a median age of 11.0 years (IQR 10.1-13.2 years), and included 6 girls at Tanner B2, 5 girls at Tanner B3, and 2 girls at Tanner B4.

### Menarche

**Figure 2:**
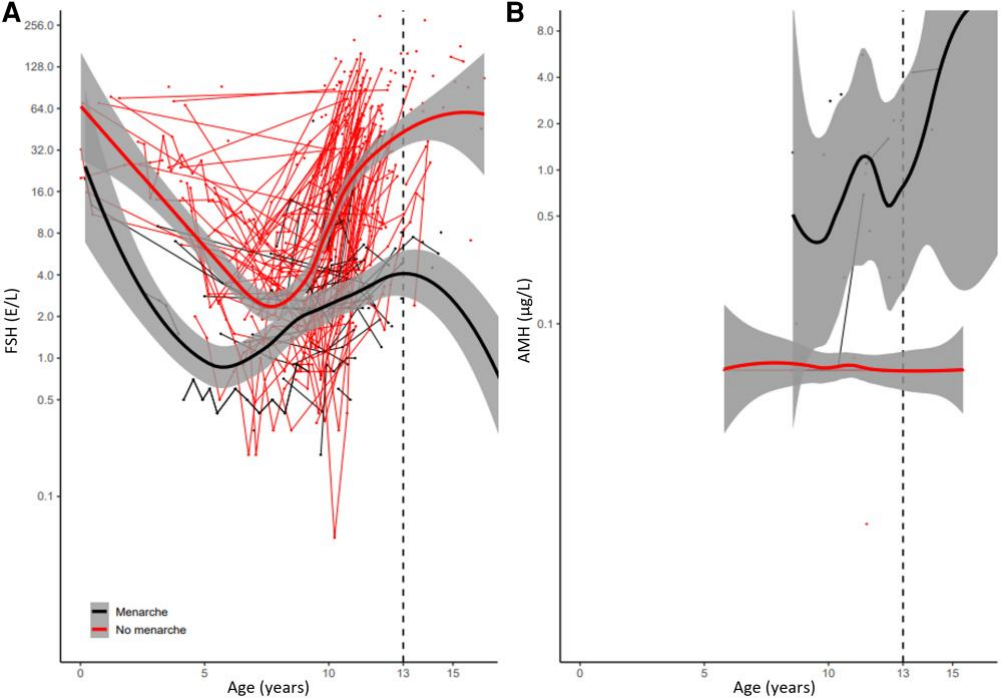
Longitudinal follicle-stimulating hormone (FSH) and antimüllerian hormone (AMH) levels and the occurrence of spontaneous menarche. Each dot represents a hormone measurement, and consecutive measurements from the same girl are connected by a line. FSH and AMH levels are presented on a logarithmic scale. The gray area represents the fitted mean and 95% CI for puberty-specific categories. No overlap between the 95% CIs suggests a statistically significant difference. The vertical dotted line at age 13.0 years represents the mean age at spontaneous menarche. In A, 67 girls with menarche and 172 girls without menarche were included. In B, 47 girls with menarche and 73 girls without menarche were included.

Spontaneous menarche occurred in 79 of 287 girls (28%) at a median age of 13.0 years (IQR 12.1-13.8 years) (see Table 1). The proportion of girls with spontaneous menarche varied between 6% in girls with a monosomy karyotype and 68% in girls with a mosaic-45,X/46,XX karyotype. Around the occurrence of menarche, 64% of the girls had at least Tanner breast development stage 4 or 5. Girls with spontaneous menarche had significantly lower FSH levels compared to girls without menarche (Fig. 2A), although this could not be confirmed during the first year of life (see Supplementary Fig. S3 [17]).

Girls with menarche had lower FSH levels while aged 8 years or younger (n = 14, maximum FSH 2.8 U/L, IQR 1.3-6.6 U/L) compared to FSH levels while aged 8 years or younger of girls without menarche (n = 53, maximum FSH 8.6 U/L, IQR 3.8-26.0 U/L). The AUC was 0.78 with the optimal cutoff identified by Youden's index was 7.2 U/L, yielding a sensitivity of 0.86, specificity of 0.58, and a Youden's index of 0.4, indicating limited discriminative ability.

Girls with spontaneous menarche had more often measurable and higher levels of AMH, compared to girls without menarche (Fig. 2B). AMH samples before age 8 years were available from 5 girls without menarche; all had levels below the detection limit. There were no AMH measurements before age 8 of girls with menarche. Girls with menarche with measurements at age 8 to 12 years had detectable AMH in 16 out of 18 girls, with a maximum AMH of 1.95 µg/L (IQR 0.62-4.38 µg/L). In contrast, only 5 out of 61 girls without spontaneous thelarche had detectable AMH (range, 0.10-0.95 µg/L). Measurable AMH at age 8 to 12 years was associated with a sensitivity of 0.88 and specificity of 0.92 for predicting spontaneous thelarche, yielding an AUC of 0.90.

At the end of study, 57 girls with menarche had continued ovarian function expressed by cyclic menstrual bleeding without elevated gonadotropins. Most of the girls with continued ovarian function had a mosaic-45,X/46,XX karyotype. In fact, 92% of girls with a mosaic-45,X/46,XX karyotype and menarche had continued ovarian function, with a median age of 15.1 years (IQR 13.4-18.9 years) at the end of the study.

### Premature ovarian insufficiency

**Figure 3:**
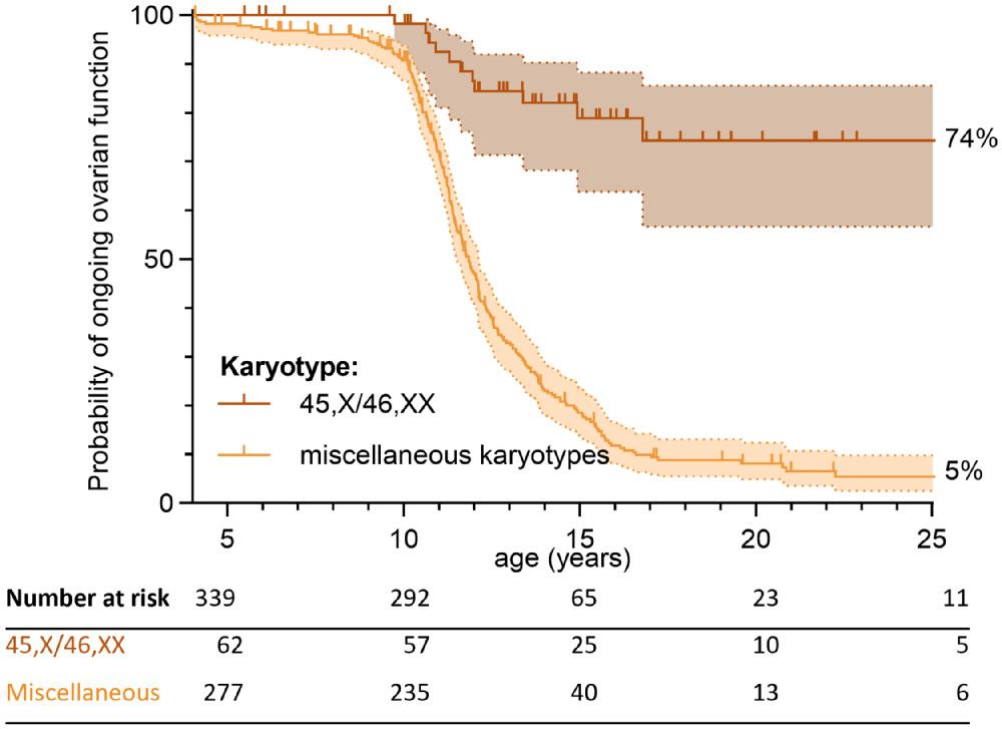
Kaplan-Meier of ongoing ovarian function in girls with Turner syndrome according to karyotype. Survival analysis (defined as ongoing ovarian function) was performed using the Kaplan-Meier method, with survival curves including error bands shown for girls with a mosaic-45,X/46,XX karyotype and girls with miscellaneous karyotype (eg, monosomy or structural aberration of X-chromosome). Censoring at last visit is indicated by tick marks. The median time of ongoing ovarian function was significantly longer in the mosaic-45,X/46,XX group (hazard ratio 6.6 for girls with miscellaneous karyotypes, 95% CI, 4.9-8.7; *P* < .01). Girls with ovarian surgery before age 12 years and without premature ovarian insufficiency were excluded (n = 17).

**Figure 4:**
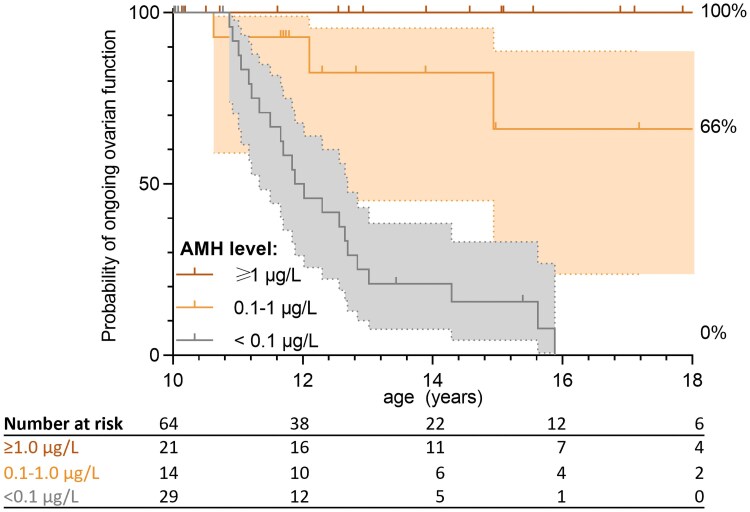
Kaplan-Meier of ongoing ovarian function in girls with Turner syndrome according to antimüllerian hormone (AMH) level. Survival analysis (defined as ongoing ovarian function) was performed using the Kaplan-Meier method, with survival curves shown for girls with spontaneous thelarche and AMH levels in 3 different groups, including error bands. Censoring at last visit is indicated by tick marks. Stratifying in groups was based on the first AMH measurement between ages 10 and 13 years.

At the end of the study, 169 girls (59%) had prepubertal POI and 48 girls (17%) POI after thelarche (see Table 1). Of 14 girls with FSH exceeding 25 U/L before age 8 years, 13 girls showed no signs of spontaneous thelarche or menarche. One girl with FSH of 25.0 U/L at age 2 years later developed breasts spontaneously (aged 11.8 years) but started with hormone replacement therapy at 15 years due to stunted puberty (Tanner B2). Figure 3 presents a Kaplan-Meier plot of ongoing ovarian function according to karyotype. Girls with mosaic-45,X/46,XX karyotype were more likely to have continued ovarian function compared to those with miscellaneous karyotypes. The hazard ratio for developing POI in the miscellaneous karyotype group was 6.6 (95% CI, 4.9-8.7; *P* < .01), compared to the mosaic-45,X/46,XX group. Most girls with miscellaneous karyotypes experienced signs of depleted ovarian reserve when they reached pubertal age, at a median age of 12.0 years. The median age at onset of POI after thelarche was 15.3 years (IQR 14.0-17.0 years) (see Table 1). Figure 4 presents a Kaplan-Meier plot of ongoing ovarian function after spontaneous thelarche, based on the first AMH measurement of a girl established at age 10 to 13 years. The hazard ratio demonstrates that girls with AMH levels between 0.10 and 1.0 µg/L were 12.8 times more likely to develop POI compared to girls with AMH levels above 1.0 µg/L (95% CI, 1.3-131.1; *P* = .03). However, the error bands around the Kaplan-Meier curves show quite some uncertainty, especially for the 0.10 to 1.0 µg/L group.

The initial steps of developing a prediction tool for the timing of POI were established using a joint model with longitudinal FSH and AMH levels. Originally intended to predict the risk of POI from birth, the tool faced limitations due to an insufficient number of measurements during the prepubertal phase. Consequently, the risk prediction model was based on all data from 4 until 30 years collected from girls with spontaneous thelarche. Hormone measurements up to age 12 years were included in the model predicting the risk on POI at age 18 years. The model included 55 girls with measurements both of FSH and AMH levels who had ongoing ovarian function at age 12. The performance of this prediction model, using data up to age 12 to predict the risk on POI after thelarche at age 18 years, was evaluated using a receiver operating characteristic curve. The AUC was 0.83. At the optimal Youden threshold, 73% of girls with POI after thelarche were correctly predicted, and of all girls with ongoing ovarian function at 18 years 83% were correctly predicted, using their measurements up to age 12. The Brier score was 0.17, suggesting a well-calibrated predictive performance.

Details on the joint model and a practical example are provided in Supplementary Fig. S4 [17].

## Discussion

This study presents the largest cohort of longitudinal hormonal parameters of girls with TS in relation to ovarian function. This study demonstrates that prepubertal elevated FSH levels likely indicate the absence of spontaneous puberty development and that longitudinal AMH levels at an early age contribute to predicting the reproductive lifespan.

Roughly, 1 in 4 girls had continued ovarian function around age 17 years, more often occurring in girls with a 46,XX cell line compared to girls with miscellaneous karyotypes. These odds are important to discuss, since for most girls the option to await natural conception or wait until adolescence for oocyte vitrification is not included in their personalized family planning options [24]. However, counseling regarding ovarian function should not be based solely on karyotype, as pregnancies have been reported in individuals with monosomy TS. Therefore, the prediction of ovarian reserve decline now also includes using hormonal data, as it directly reflects ovarian function [4, 25, 26].

Similar to other studies, gonadotropins showed a pattern characterized by a brief increase during minipuberty, followed by low levels between ages 6 and 10 years, and a subsequent increase thereafter [8, 10]. Some studies have suggested that low gonadotropins during mid-childhood indicate the occurrence of spontaneous thelarche and menarche [8, 9], while others have stated that gonadotropins remained low even in cases with an absence of spontaneous puberty development [10]. Our study demonstrated that prepubertal low FSH is not predictive of the occurrence of spontaneous puberty. This is likely influenced by the quiescent hypothalamus-pituitary-gonadal axis. Elevated prepubertal FSH is an expression of depletion of the ovarian reserve in the short term and therefore a predictor that spontaneous thelarche and menarche are unlikely.

In cases with low mid-childhood gonadotropins, AMH levels are presumed to be more representative of ovarian reserve because it is produced by the cohort of growing follicles [26, 27]. AMH levels distinguish between girls with ongoing ovarian function during adolescence and girls with imminent POI in the near future [28]. This is supported by studies presenting an association between AMH level and the number of follicles in the ovary, both during prepuberty and puberty [4, 29]. However, exceptions are reported with detectable AMH and the absence of follicles [4], or absence of spontaneous puberty [16] and, in contrast, AMH below the detection limit and spontaneous onset of puberty [13, 15, 16, 30]. Consequently, establishing a definitive AMH cutoff level to accurately predict remaining ovarian reserve, and therefore the likelihood of spontaneous puberty, remains challenging [12]. This is even more complex due to the high intertest and intraindividual variations in AMH levels [31]. The implementation of repetitive AMH measurements is likely to contribute to a better prediction of the remaining ovarian reserve. Inhibin B was not often measured during the prepubertal phase and was mostly below the detection limit. Therefore, inhibin B is likely of limited value in predicting POI at an early age. Around pubertal age detectable levels of inhibin B could be of added value in discriminating between girls with POI or with ongoing ovarian function; however, the supporting evidence remains limited [10, 32].

Besides childhood hormone levels, minipuberty could provide a window of opportunity to gain insight into ovarian reserve [33]. During minipuberty, shortly after birth, the hypothalamus-pituitary-gonadal axis is briefly activated because of the loss of maternal and placental hormones [34]. Johannsen et al (2018) [35] described elevated gonadotropins during minipuberty in 4 girls with TS compared to girls without TS. Sawyer et al (2025) [36] described that an LH level greater than 0.5 U/L or FSH level greater than 37.4 U/L before age 3 years predicted the absence of spontaneous thelarche, with accuracies of 94% and 97%, respectively. In the present study, minipuberty hormone levels (<4 years) were available for 18 girls with TS and known puberty development, and several cases without spontaneous thelarche had FSH levels lower than 37.4 U/L. However, our study confirms that elevated gonadotropins during minipuberty are correlated to an absence of spontaneous pubertal development. If spontaneous thelarche occurred in girls with high FSH during minipuberty, the ovarian reserve was depleted before the occurrence of menarche.

A reliable prediction model might be achievable in the future with the availability of minipuberty and prepubertal gonadotropin and AMH measurements. Based on the limited data in our registry, our prediction model is not yet reliable, though it shows promise with the accumulation of more data. An updated prediction model would enhance the counseling on family planning options and facilitate timely referral for fertility preservation.

To the best of our knowledge, this is the largest cohort study, including longitudinal hormonal data from multiple TS expertise centers. However, despite the large number of included individuals, the actual sample size underlying the various statistical measures remains limited. Our study includes 3 important limitations. First, it could be argued that these results are not easily extrapolated to all girls with TS, since the karyotype in this cohort was based on analysis of lymphocytes only. It is well documented that a comprehensive analysis of the genotype in 2 cell lines is advisable [25, 37]. Still, genotype does not necessarily reflect ovarian function, whereas hormone levels directly indicate ovarian activity. Second, no data on the time of the day or phase in the menstrual cycle at blood sampling were available. Therefore, the interpretation of gonadotropins during onset of puberty may be influenced by the circadian rhythm with peak values in the early morning [38], and gonadotropin levels are different during the several phases of the menstrual cycle. And last, the diagnosis of prepubertal POI was not always based on FSH greater than 25 U/L, but because of an initiation of estradiol for puberty induction (3.4% of 264 prepubertal POI measurements). However, in all these measurements, FSH greater than 25 U/L before age 5 years (n = 1), undetectable AMH (n = 1), or age above 12 years without spontaneous breast development was observed (n = 7), leading to the initiation of estradiol suppletion. This approach is in line with the TS guidelines, which recommend promoting breast development around ages 11 to 12 years. Ideally, the diagnosis of POI should be based on 2 consecutive measurements of FSH greater than 25 U/L at least 1 month apart. However, this approach was not feasible for this retrospective cohort study.

Girls with TS deserve counseling on their personal ovarian function and the expected reproductive lifespan to make a timely and informed decision on family planning options. Although genotype is a predictive characteristic, repeated measurements of AMH, including during early infancy and childhood, may offer a more precise and dependable prediction of ovarian function over time. A reliable prediction model in the future would be an important goal for advancing counseling on personalized family planning options.

## Data Availability

Datasets generated during and analyzed during the current study are not publicly available but are available from the corresponding author on reasonable request.

## Abbreviations

AIC: Akaike information criteria
AMH: antimüllerian hormone
AUC: area under the receiver operating characteristic curve
FSH: follicle-stimulating hormone
IQR: interquartile range
LH: luteinizing hormone
LLOD: lower limit of detection
OTC: ovarian tissue cryopreservation
POI: premature ovarian insufficiency
TS: Turner syndrome
